# A comparative cross-sectional study on sleep quality in patients with a history of differentiated thyroid carcinoma and its correlation with quality of life

**DOI:** 10.1007/s12020-020-02591-z

**Published:** 2021-02-03

**Authors:** Marsida Teliti, Eleonora Monti, Martina Comina, Lucia Conte, Lara Vera, Stefano Gay, Giorgia Saccomani, Diego Ferone, Massimo Giusti

**Affiliations:** 1 Endocrinology Unit, IRCCS Ospedale Policlinico San Martino, Genoa, Italy; 2 Endocrinology Unit, Department of Internal Medicine & Medical Specialties (DiMI), University of Genoa, Genoa, Italy; 3 Centro Diagnostico Priamar, Savona, Italy

**Keywords:** Thyroid cancer, Sleep quality, PSQI, ISI, QoL, ThyPRO

## Abstract

**Purpose:**

To evaluate sleep quality in differentiated thyroid carcinoma (DTC) patients and correlate sleep disturbances with quality of life (QoL).

**Methods:**

119 DTC patients were enrolled (DTC group). The Pittsburgh Sleep Quality Index (PSQI) and the Insomnia Severity Index (ISI) inventories were administered. The Thyroid-specific Patient-Reported Outcome (ThyPRO) questionnaire, the Billewicz scale (BS) and an ad-hoc visual analogic scale (VAS) were used to measure QoL and subjective therapy-related complaints. The same examinations were conducted in 53 subjects (control group) who had undergone surgery for benign thyroid pathology.

**Results:**

L-T4 dosages and TSH levels differed between the groups. BS and VAS scores were comparable. PSQI documented a similar percentage of poor sleepers in the DTC (74%) and control (62%) groups. ISI showed no difference in subjects without clinically significant insomnia: DTC (43%) and controls (48%). ThyPRO showed significantly worse scores in DTC than control subjects. In DTC patients, PSQI (*P* = 0.002) and ISI (*P* = 0.04) correlated significantly with age. In control subjects, TSH displayed a significant positive association with PSQI (*P* = 0.02) and ISI (*P* < 0.05). The ThyPRO general score correlated significantly with PSQI in DTC patients. In both groups, ISI correlated significantly with several ThyPRO scales and the ThyPRO general score. “Anxiety” and “emotional susceptibility” were the scales most significantly related with PSQI and ISI.

**Conclusion:**

In disease-free DTC patients and subjects who undergo thyroid surgery for benign pathology, abnormal sleep components and insomnia are similar. The ThyPRO questionnaire closely reflects sleep disturbances in all subjects. Recognising and treating sleep disturbances might improve QoL.

## Introduction

There is an association between thyroid function and sleep. It is well known that the circadian rhythm and sleep-wake state can affect the secretion of TSH-thyroid hormones [1–3]. Patients with hyperthyroidism or hypothyroidism experience sleep disturbances, such as difficulty initiating and maintaining sleep, or reduced or even increased slow-wave sleep [4, 5]. Subjects with sub-clinical hypothyroidism suffer more often from weakness and reduced muscle strength; this may result in insufficient strength for regular ventilation or the patency of upper airways [6], which are risk factors for sleep apnoea syndrome [7]. The hypothalamic–pituitary–thyroid axis is under the control of the supra-chiasmatic nucleus pacemaker [8]. Disruption of circadian rhythms has been recognised as a perturbation of the cell-cycle progression, and abnormal expression of circadian clock genes in differentiated thyroid cancer (DTC) has been suggested [8].

Several studies have documented poor sleep quality in patients with cancer, the incidence of sleep disturbance in oncologic patients being at least twice that found in the general population [9–13]. Sleep disorders in these patients can be debilitating, resulting in poor quality of life (QoL). In this regard, Pelttari et al. [14] found that, over long-term follow-up, overall QoL in DTC patients was comparable to that of the general population, but that thyroid cancer survivors reported a significant impairment in the single dimensions of sleep, speech and distress. This finding highlights the importance of assessing sleep disturbance in long-term survivors of DTC. To date, only a few studies [15–19] have examined the quality of sleep in DTC patients and to our knowledge only the study by Pelttari et al. attempted to correlate sleep and QoL in these patients.

In 2011, we published a case-controlled longitudinal study on QoL in DTC patients followed up for 5 years, to whom the self-rated Kellner symptom questionnaire was administered in association with a semi-structured psychiatric interview. The study documented a wide variation in DTC patients’ perception of their illness, and psychological evaluation improved during long-term follow-up [20].

Poor sleep quality has been described during thyroid cancer treatments due to very high levels of somatic anxiety and worries about prognosis [16]. Little is known about the sleep disturbances in DTC patients after long-term follow-up. The aim of the present study was to investigate the quality of sleep in a large group of male and female DTC patients, generally with a favourable prognosis, undergoing periodic follow-up examinations. Scores obtained from two subjective inventories for sleep evaluation were correlated with clinical data and the self-reported ThyPRO questionnaire. The primary objective of the study was to evaluate sleep in DTC patients and to compare their scores with those of subjects who had undergone thyroid surgery for benign pathology. In addition, a secondary objective was to identify clinical and QoL-related determinants of sleep disturbances.

## Materials and methods

### Study design and subjects

A comparative cross-sectional study was implemented. Patients were recruited from our Endocrine Unit during their 2019 periodic examination. The exclusion criteria were: (1) presence of severe cognitive impairments or severe psychiatric disorder; (2) presence of a sleep disorder other than insomnia (e.g., sleep apnoea); (3) inability to read and understand Italian.

Disease-free DTC patients (DTC group) were invited to participate in the survey if they were aged 18–85 years and had completed treatment for thyroid cancer at least 1 year previously. On the basis of the functional sensitivity of the thyroglobulin (Tg) assays, DTC patients with undetectable Tg levels, negative anti-Tg antibodies (TgAb) and negative neck sonography were considered disease-free. Low-risk DTC patients without a history of radioiodine ablative treatment after surgery in whom Tg levels were low but detectable or stable over time and neck US imaging was negative were also considered to be free from disease.

In the same period, inventories were administered to consenting subjects (control group) aged 18–85 years who had undergone thyroid surgery (92.5% total thyroidectomy) for a benign pathology (goitre, hyperthyroidism, indeterminate cytology and suspicious calcitonin level) at least 1 year previously.

### Protocol

The PSQI and ThyPRO questionnaires were administered to all patients 4 weeks before the time of scheduled examinations. Clinical examination comprised history, evaluation of current therapies, physical examination, neck sonography (MyLab Five equipped with a 7.5–10.0 MHz linear probe; Esaote, Genoa, Italy), blood tests, collection of previously compiled questionnaires, and compilation of ISI and BS inventories. DTC and control subjects were asked to complete an ad hoc visual analogic scale (VAS). All patients provided written consent by filling in the Policlinico San Martino consent form entitled “*trattamento dei dati personali e sensibili”* (“handling of personal and sensitive data”) in accordance with EU regulation 2016/679. Approval for the study was obtained from the Liguria Ethics Committee.

### Survey measurements

The Italian version of the PSQI, a validated self-rated questionnaire that assesses sleep quality and sleep disturbance over a 1-month period, was used [21]. The 19 items in the index generate seven component scores that reflect problems in the following areas: subjective sleep quality, sleep latency, sleep duration, habitual sleep efficiency, sleep disturbances, use of sleep medications, and daytime dysfunction. The scores for these seven components are computed in order to obtain a global score ranging from 0 to 21 points. In accordance with Buysse et al. [21], a cut-off value >5 was adopted in order to distinguish poor sleepers from good sleepers. The ISI is a 7-item self-report questionnaire that assesses the nature, severity and impact of insomnia [22]. The usual recall period is “the last month” and the dimensions evaluated are: difficulty of sleep onset, sleep maintenance and problems of early morning awakening, sleep dissatisfaction, interference of sleep difficulties with daytime functioning, noticeability of sleep problems by others, and distress caused by sleep difficulties. A 5-point scale is used to rate each item yielding a total score ranging from 0 to 28. QoL was measured by means of the Italian version of ThyPRO [23]. The Italian version of the Billewicz scoring index [24, 25] was used to assess disease-specific morbidity (or inadequate L-T4 treatment). An extensive description of ThyPRO questionnaire and Billewicz scoring index has been previously provided [26]

Subjective complaints related to medical therapy were registered by means of ad hoc VAS: a score of 0 indicating no complaints and 10 the worst degree of complaint due to ongoing medical therapies.

### Laboratory evaluation

Free-T4 (f-T4) and TSH levels were measured by means of electro-chemiluminescence immunoassay, optimised on the Cobas platform (Roche Diagnostics, Milan, Italy); reference ranges are 12.0–22.0 pmol/L for f-T4 and 0.3–4.2 mIU/L for TSH. TSH functional sensitivity is 0.03 mIU/L, with intra- and inter-assay imprecision of 3% and 7%, respectively. Tg and TgAb were evaluated as previously reported [27].

### Statistical methods

The Kolmogorov–Smirnov test was applied in order to check the normality of continuous variables. Categorical variables were described as percentages, and continuous variables as mean, SEM, median, and 25th–75th interquartile range (IQR) values. Continuous variables were not normally distributed. The Mann–Whitney test was used. Regarding categorical variables, percentages were compared by means of the Chi-square or Fisher exact test. Relationships among variables were sought by applying Spearman coefficient of correlation (rS). Multiple linear regressions were performed to model the relationship between the dependent variables PSQI general score and ISI and the following independent variables: age, years since surgery, BMI, L-T4 dosages, TSH, f-T4, BS and VAS. *P* values of less than 0.05 were considered statistically significant. All statistical analyses were performed by means of GraphPad 8.4.0 software (San Diego, CA, USA). Data collection and subsequent analysis were performed in compliance with the Helsinki Declaration. The self-rated questionnaires were deemed assessable when filled in >95% correctly; this explains the variation in the number of pairs in the statistical analysis carried out.

## Results

### Clinical data

**Fig. 1:**
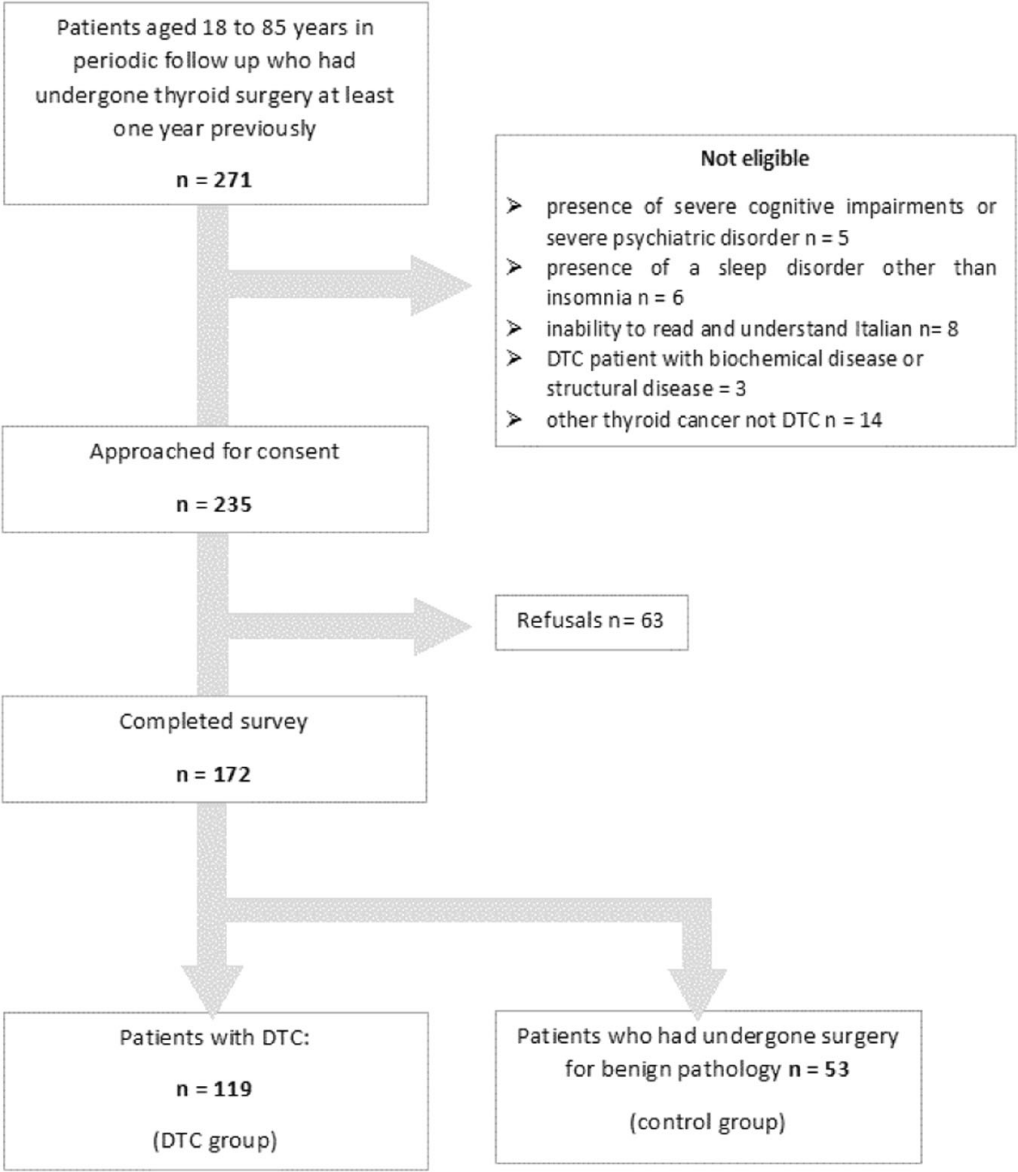
Flow diagram of the study population

Out of 235 eligible patients, 172 subjects (73%) were enrolled in the study. Figure 1 shows the study population flow diagram. One-hundred and nineteen DTC patients (DTC group) consented to participate in the evaluation of several components of sleep by means of the PSQI and ISI. These sleep inventories were then correlated with the self-reported ThyPRO Questionnaire. All but five subjects had a history of total thyroidectomy (95.6%). DTC histology was: papillary, follicular variant of papillary, follicular, and others (*n* = 1 follicular variant of papillary in struma ovarii, *n* = 1 Hurtle, and *n* = 3 cancer of unknown malignant potential) in 95, 15, 4 and 5 subjects, respectively. Retrospective tumour reclassification, according to the 8th edition of the American Joint Committee on Cancer, was available in 116 subjects. A total of 89% of DTC were at stage 1, while the remainder were at stage 2 (10%) or stage 3 (1%). In the same period, inventories were administered to 53 consenting subjects (control group) who had undergone thyroid removal for benign pathology.

Table 1 reports the demographic and clinical data of the study and control groups. All subjects were on suppressive or substitutive treatment with L-T4, and any psychotropic drugs taken were recorded during the examination.

**Table 1:** Some demographic and clinical data on the DTC and control groups

	DTC group	Control group	*P*
Number of subjects	119	53	
Age (years) mean ± SD	61.5 ± 13.1 (63; 51–72)	63.6 ± 14.3 (68; 56–73)	0.15
Gender F/M (number)	92/30	47/6	0.07
Years since surgery	9.9 ± 0.7 (8; 4–14)	10.0 ± 1.0 (8; 5–12)	0.82
DTC stage (number) 1	104	–	
2	11	–	
3	1	–	
Unknown	3	–	
RAI ablation (%)	71	–	
BMI (kg/m^2^)	26.8 ± 0.5 (25.9; 23.0–29.3)	26.1 ± 0.7 (25.2; 22.9–29.2)	0.56
Normal weight (%)	44	48	0.39
Overweight (%)	35	31	
Obesity (%)	21	21	
L-T4 dosages (kg/b.w./day)	1.54 ± 0.03 1.49 (1.29–1.71)	1.40 ± 0.07 1.35 (1.15–1.57)	0.01
TSH (mIU/L)	1.17 ± 0.26 0.36 (0.09–1.15)	2.62 ± 0.44 1.56 (0.83–3.02)	<0.0001
f-T4 (pmol/L)	19.0 ± 0.6 18.5 (16.0–21.4)	18.1 ± 0.6 18.0 (14.7–20.5)	0.23
Tg (µg/L)	0.62 ± 0.18 0.2 (<0.04–0.2)	n.d.	
TgAb positive (%)	0.8	n.d.	
Psychotropic drugs (%)	18	19	0.32

Data are reported as mean ± SEM if not otherwise stated, and in brackets as median plus IQR

*n.d.* not done

On entry to the study, both groups of subjects did not differ significantly in terms of age, male/female ratio and time since surgery. Median BMI was similar between the groups, as were the percentages of normal-weight, overweight (BMI > 25 kg/m^2^) and obese (BMI > 30 kg/m^2^) patients. Owing to their different diagnoses, the two groups differed in terms of therapeutic strategies, L-T4 dosages and TSH, but not f-T4 levels. Most DTC patients had a very low tumour stage on diagnosis and were disease-free at the time of the study. While the percentage of subjects taking psychotropic drugs was similar in both groups, a significantly higher percentage of DTC subjects (96%) than control subjects (84%) were taking other drugs (*P* = 0.02); this was mainly due to a more generalised administration of cholecalciferol (71% vs 54%) and metformin (26% vs 4%). Nevertheless, the frequency of diabetes mellitus was similar (<2%) in both groups. VAS scores were similar (*P* = 0.40) in the DTC (2.6 ± 0.2; median 1.0, IQR 1.0–5.0) and control (2.0 ± 0.3; 1.0, 1.0–3.0) groups. No difference (*P* = 0.83) in BS score was noted between the DTC (2.0 ± 0.2; median 2.0, IQR 1.0–3.0) and control (2.0 ± 0.2; 2.0, 1.0–3.0) groups.

### Sleep and QoL measures

**Fig. 2:**
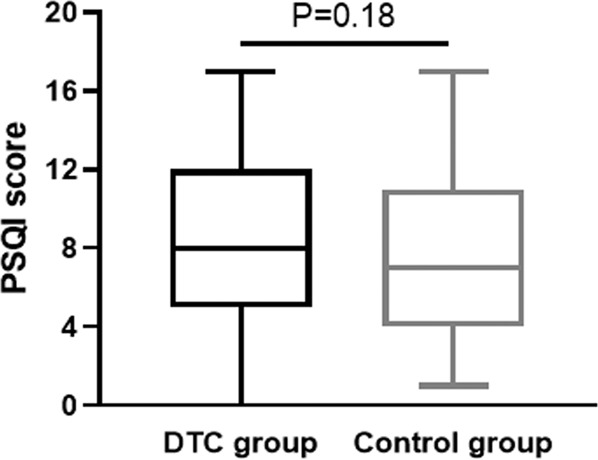
Median and IQR of PSQI scores in the DTC and control groups. Whiskers indicate the PSQI range

**Fig. 3:**
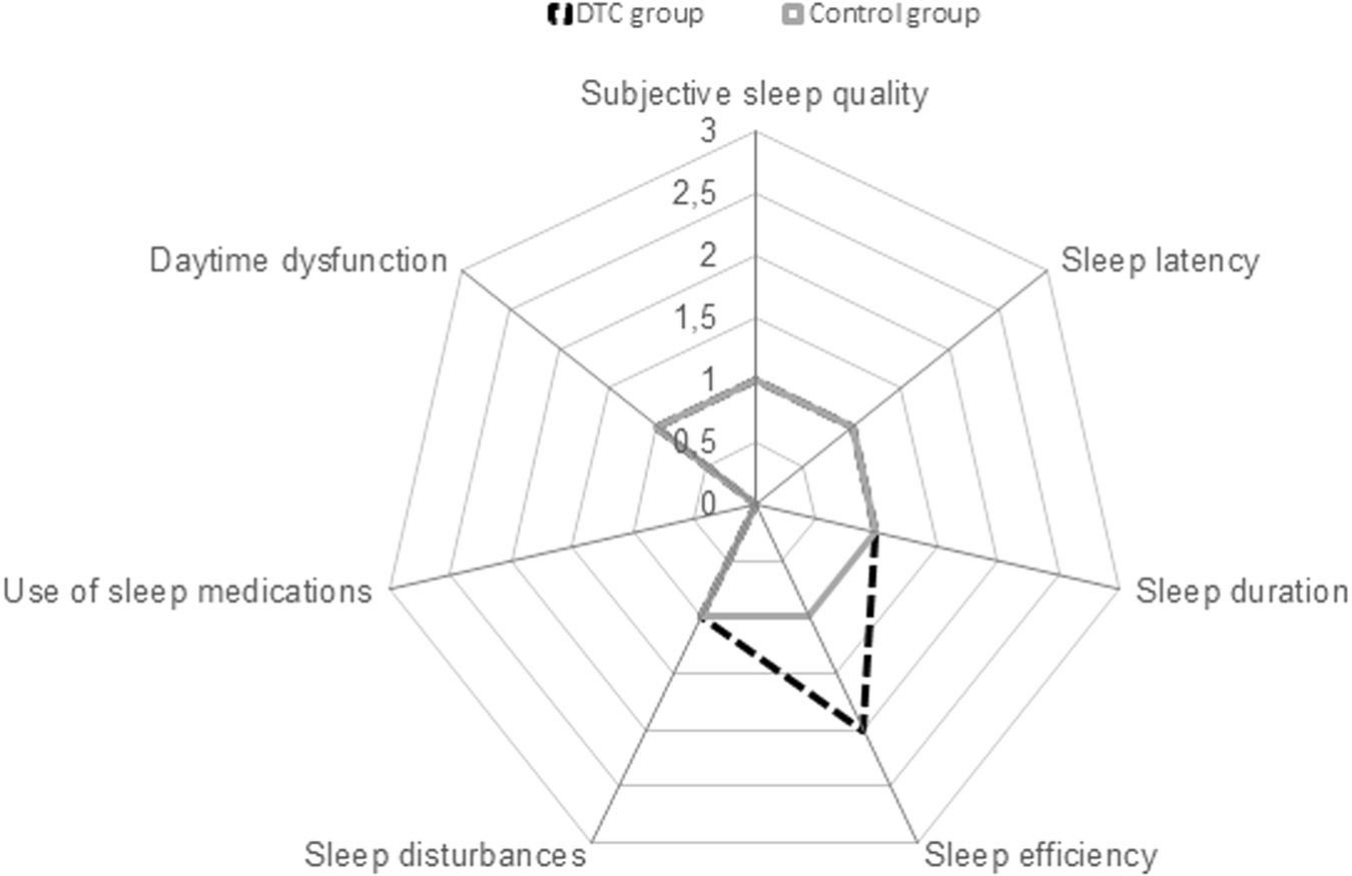
Radar plot of median PSQI determinants evaluated in the DTC and control groups

Figure 2 summarises the median and IQR of PSQI scores in the DTC and control groups. No significant difference in scores was noted between the groups. The percentage of poor sleepers was 74% in the DTC group and 62% in the control group (*P* = 0.18). The radar-plot showing the components of the PSQI score is reported in Fig. 3. The scores of all components were sometimes slightly higher in DTC subjects than in controls, but only “Sleep efficiency” was significantly different (*P* = 0.04), while the difference in “Daytime dysfunction” approached significance (*P* = 0.06) (Fig. 3). Median ISI scores were 7.0 (IQR 4–11) in the DTC group and 10.0 (3–14) in the control group (*P* = 0.52). The percentage of subjects with no clinically significant insomnia (ISI score: 0–7) was not significantly (*P* = 0.82) different between DTC (43%) and control (48%) subjects.

**Fig. 4:**
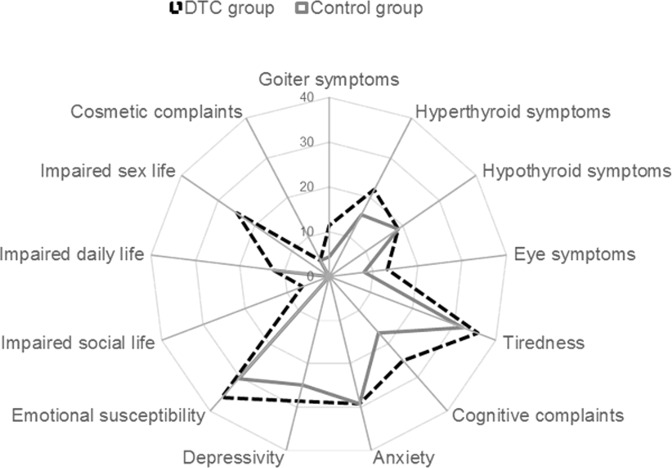
Radar plot of median ThyPRO scores evaluated in the DTC and control groups

Figure 4 summarises the median ThyPRO scores in both groups. Worse scores were noted in the DTC group than in the control group on several scales [goitre symptoms (*P* = 0.05), eye symptoms (*P* = 0.07), cognitive problems (*P* = 0.04) and impaired sex life (*P* = 0.01)]. Median overall QoL, evaluated by means of the last single item, dubbed “general score”, was 1.0 (0–2) in DTC and 0.0 (0–1) in control subjects (*P* = 0.002).

### Determinants of sleep

#### Bivariate correlation analyses

In the DTC group, the variable PSQI general score was significantly related only with age (*P* = 0.002) (Table 2). PSQI was not related with the other clinical variables nor with BS or VAS scores in either group (Table 2). There was a significant negative correlation between the ISI inventory and age in the DTC group (*P* = 0.04) and between ISI and VAS scores in the control group (*P* = 0.02) (Table 3). No other significant correlation was noted between ISI and other clinical variables and scales in either the DTC or control group (Table 3). The ThyPRO general score was significantly related to PSQI in the DTC group (*n* = 109; rS 0.23, *P* = 0.02) but not in the control group (*n* = 47, rS 0.26, *P* = 0.08), while ISI was significantly related with the ThyPRO general score in both the DTC (*n* = 86; rS 0.27, *P* = 0.01) and control (*n* = 20; rS 0.46, *P* = 0.04) groups. Tables 2 and 3 report the correlation between each scale on the ThyPRO questionnaire and the PSQI and ISI inventories in both the DTC and control groups. PSQI displayed a significant positive correlation with all ThyPRO scales in the DTC group and with 10/13 scales in the control group (Table 2). The ISI inventory was positively related with 9/13 and 8/13 scales in the DTC and control groups, respectively (Table 3). The scales “anxiety” and “emotional susceptibility” were those most significantly related with PSQI and ISI in both groups (Tables 2, 3).

**Table 2:** Bivariate correlation between PSQI general score and clinical parameters, BS, VAS, and ThyPRO scales

	DTC group			Control group		
	No. pairs	rS	*P*	No. pairs	rS	*P*
Age (years)	118	0.28	0.002	53	0.10	0.46
Years since surgery	118	0.10	0.27	53	0.07	0.61
BMI (kg/m^2^)	118	0.07	0.45	52	−0.05	0.73
L-T4 dosages (kg/b.w./day)	117	−0.14	0.12	53	0.02	0.90
TSH (mIU/L)	118	0.01	0.94	53	0.20	0.15
f-T4 (pmol/L)	115	0.06	0.55	52	0.15	0.27
BS	115	0.14	0.14	44	0.03	0.83
VAS	92	0.12	0.27	22	−0.33	0.13
Goitre symptoms	113	0.33	0.0003	50	0.31	0.03
Hyperthyroid symptoms	113	0.31	0.001	50	0.47	0.001
Hypothyroid symptoms	116	0.31	0.001	48	0.25	0.08
Eye symptoms	115	0.40	<0.0001	50	0.10	0.48
Tiredness symptoms	116	0.45	<0.0001	48	0.31	0.03
Cognitive problems	116	0.32	0.0004	49	0.31	0.03
Anxiety	113	0.52	<0.0001	49	0.44	0.002
Depressivity	114	0.34	0.0002	47	0.36	0.01
Emotional susceptibility	115	0.38	<0.0001	48	0.42	0.003
Impaired social life	113	0.35	0.0001	49	0.49	0.0003
Impaired daily life	110	0.30	0.001	48	0.51	0.0002
Impaired sexual life	102	0.22	0.03	41	0.54	0.0003
Cosmetic complaints	111	0.20	0.04	48	0.26	0.07

**Table 3:** Bivariate correlation between ISI and clinical parameters, BS, VAS, and ThyPRO scales

	DTC group			Control group		
	No. pairs	rS	*P*	No. pairs	rS	*P*
Age (years)	92	−0.21	0.04	23	−0.24	0.28
Years since surgery	92	0.03	0.76	23	0.06	0.78
BMI (kg/m^2^)	92	−0.12	0.18	23	−0.26	0.24
L-T4 dosages (kg/b.w./day)	92	0.11	0.30	23	0.15	0.51
TSH (mIU/L)	92	−0.07	0.48	23	0.21	0.35
f-T4 (pmol/L)	92	0.06	0.55	23	0.09	0.69
BS	90	0.14	0.18	21	−0.16	0.48
VAS	91	0.13	0.21	21	−0.50	0.02
Goitre symptoms	88	0.31	0.004	21	0.23	0.31
Hyperthyroid symptoms	88	0.42	<0.0001	21	0.62	0.003
Hypothyroid symptoms	91	0.44	<0.0001	19	0.13	0.60
Eye symptoms	90	0.37	0.0004	21	0.33	0.14
Tiredness symptoms	91	0.40	<0.0001	19	0.41	0.08
Cognitive problems	91	0.32	0.002	20	0.51	0.02
Anxiety	89	0.43	<0.0001	20	0.64	0.002
Depressivity	89	0.33	0.002	19	0.60	0.01
Emotional susceptibility	91	0.48	<0.0001	19	0.64	0.003
Impaired social life	89	0.13	0.22	20	0.20	0.39
Impaired daily life	87	0.18	0.09	19	0.64	0.003
Impaired sexual life	81	0.16	0.20	18	0.62	0.01
Cosmetic complaints	87	0.13	0.20	20	0.62	0.004

#### Multiple linear regression analyses

In the multiple regression analysis with PSQI general score as the dependent variable, the independent variables age, years since surgery, BMI, L-T4 dosages, TSH, f-T4, BS and VAS did not attain significance in the DTC group (overall model fit *R*^2^ = 0.11). In the control group, the variable PSQI (overall model fit *R*^2^ = 0.67) displayed a significant positive association with both BS (*β* = 1.85, SE = 0.69, *t* value = 2.67, *P* value = 0.02) and TSH (*β* = 2.20, SE = 0.77, *t* value = 2.87, *P* value = 0.02) and a significant negative association with VAS (*β* = −1.74, SE = 0.63, *t* value = 2.76, *P* value = 0.02). In the multiple regression analysis with ISI as the dependent variable, the variable VAS had a significant positive association (*β* = 0.54, SE = 0.26, *t* value = 2.08, *P* = 0.04) in the DTC group (overall model fit *R*^2^ = 0.17) and a significant negative association (*β* = – 2.44, SE = 0.88, *t* value = 2.78, *P* = 0.02) in the control group (overall model fit *R*^2^ = 0.60); the dependent variable ISI also displayed a positive association (*β* = 2.42, SE = 1.07, *t* value = 2.27, *P* < 0.05) with TSH in the control group.

## Discussion

Conducted on a cohort of DTC patients, most of whom were deemed “cured” after primary treatments, our study utilised self-reported inventories constructed to evaluate sleep components. We found that these DTC patients suffered the same degree of sleep impairment as that observed in control subjects who had undergone thyroid removal for benign pathology. Furthermore, our data showed that, a long time after primary treatments, cumulatively more than 60% of subjects with controlled post-surgical hypothyroidism suffered from sleep disturbances. This finding is in line with the fact that quite a large percentage of DTC and control subjects (18–19%) were taking psychotropic substances, in addition to several other drugs.

We hypothesise that trend in sleep quality changes following diagnosis. A poorest sleep quality due to both physiological and psychological stressors associated with the diagnosis and the primary treatments affects DTC patients during the early stage of the disease. Meanwhile, after several years, DTC patients successfully treated and subjects who had undergone surgery for benign thyroid pathology only deal with the effect of L-T4 replacement therapy.

Sleep disturbances, mainly sleep deprivation and disruptions in circadian synchronisation, are common in modern society, as a result of occupational and personal pressures [28, 29]; indeed, disturbed sleep is the most frequent health complaint encountered [30]. In the adult general population, the incidence rate of disturbed sleep is reported to vary widely from 9 to 33% [22, 29–31]. Sleep quality has a major impact on QoL in cancer patients, in whom the incidence rate of disturbed sleep has been reported to range from 30 to 93% [9–11].

Very few literature data on sleep quality in thyroid cancer patients have been reported. He et al. [16], evaluated the subjective quality of sleep by means of the PSQI in 162 DTC patients at the moment of radioiodine ablation of thyroid remnants about 1 month after surgery, and compared these data with those collected from 84 patients 1 month after thyroid removal for benign nodules. Poor sleepers (PSQI score >5) were found in 54% and 33% of DTC and non-DTC patients, respectively, 1 month after surgery. On re-testing, the percentage of poor sleepers in the DTC group was seen to have increased to 71% after ablation, and reached 79% in the sub-group of patients in whom imaging documented metastatic disease [16].

It is difficult to compare the present data with those of He et al. as the time of PSQI administration differed greatly. Moreover, their study did not report hormonal data, and it can be assumed that, 1 month after surgery, non-DTC patients were on L-T4 at dosages that were not yet personalised, and that DTC patients were in severe hypothyroidism, as radioiodine ablation is performed after triiodothyronine withdrawal. In addition, patient age, which markedly modulates sleep duration [32, 33], was different in the two studies, the mean age being 40 years in that of He et al. [16]. and >60 years in ours. Moreover, we can speculate that sleep disturbances increase after surgery with chronological age more than as a result of oncologic disease per se.

Recently, Jung and Visovatti [17] compared female thyroid cancer survivors with healthy women, and observed that sleep problems and fatigue symptoms were predicted by lower perceived cognitive effectiveness, worse cognitive performance and reduced QoL. Berti et al. [18] suggest that, while sleep analysis should be included in thyroid cancer research and in clinical practice, the PSQI may not be sufficient to identify patients with poor sleep quality who need additional support to deal with the side-effects of disease and its treatment. In a population of 142,933 women, who were free from cancer at the baseline and were evaluated for insomnia, Luo et al [19]. reported a <1% incidence of thyroid cancer after an 11-year follow-up. After adjustment for potential confounders, non-obese women with greater insomnia scores showed a significantly higher risk of thyroid cancer than did women with low scores.

In our study, we also employed the ISI inventory to determine the percentage of clinical insomnia, though without finding any significant difference between DTC (43%) and control (48%) subjects. These data do not seem to support the hypothesis of Lou et al. [19] that insomnia can be a facilitator condition in the appearance of thyroid cancer. In addition, in a total of 297,185 retired people (age range 50–71 years) who filled in a self-reported questionnaire regarding their length of sleep, Gu et al. [34] observed a significant association only between stomach cancer among men and short duration of sleep. However, once they had removed BMI and diabetes from their multivariate analysis, they found that, in men, the incidence of other kinds of cancer, including thyroid cancer, tended to increase (about 1.5–2.0-fold increase) as the duration of sleep decreased. This last finding was not explained by the authors [34] and further studies will be necessary to evaluate the risk of thyroid cancer in a condition of short sleep duration.

While diabetes mellitus was encountered in very few of our patients, overweight and obesity were observed in more than half of the subjects, without significant differences between the groups. No significant correlation emerged between PSQI or ISI scores and BMI on either bivariate correlation or multiple regression analysis. Although sleep disturbance, mainly daytime sleepiness, is a well-known phenomenon, being reported in about 60% of subjects with obesity and obstructive sleep apnoea syndrome [35, 36], the 21% of obesity in our cohorts of subjects does not seem to explain the cumulative incidence of sleep disturbance after thyroidectomy.

Thyroid function is thought to be an important determinant of sleep quality. In 628 males aged >65 years, Akatsu et al. [37] analysed the association between thyroid function and objective sleep quality by using a “sleep watch” actigraph worn on the non-dominant wrist. In this population, which differed from our sample in terms of gender distribution, median age, and median TSH levels, the percentages of sub-clinical hyperthyroidism and sub-clinical hypothyroidism were 2% and 6%, respectively. In this sub-set of patients with TSH outside the normal range, the percentages of poor sleepers (sub-clinical hyperthyroidism: 46%; sub-clinical hypothyroidism: 50%) did not differ from that reported in euthyroid subjects (40%) [37].

Kramer et al. [38] observed that the short-term administration of supra-physiologic dosages of L-T4 in healthy subjects did not change sleep architecture. On the other hand, in a recent Chinese study [39] in which PSQI was administered to a large number of subjects, the proportion of poor sleepers was higher in the sub-clinical hypothyroid subjects than the euthyroid subjects. Finally, Kadoya et al. [40] reported that, in patients with cardiovascular risk, poor sleep quality was associated with an increase in the macro-molecular form of TSH; however, this phenomenon has been observed only in a small portion of DTC patients [41].

Most studies have found that DTC patients suffer an impairment of their QoL [14, 20, 26, 42–45], as do patients with tumours of other sites [46]. The ThyPRO, a specific inventory validated for the assessment of QoL in patients with benign thyroid disease [23], was used by Massolt et al. [45] in patients with a history of DTC. In our recent study [26] of 123 DTC patients, QoL was analysed by means of all the scales of the ThyPRO questionnaire; the DTC patients and 192 controls subjects had similar scores on all but one scale. Indeed, only scores on the hyperthyroid symptoms scale were significantly higher in DTC patients than controls [26]. In the present study, ThyPRO documented worse perceptions of illness and significantly higher scores on several test scales in the DTC group. Akatsu et al. [37] used the Short-Form 12 inventory to identify depression (4%) and anxiety (5%) in their study population under sleep evaluation, but they did not attempt to correlate subjective and objective sleep parameters with the data from the psychometric test. Demartini et al. [47] used the Hamilton-D test in 123 subjects with sub-clinical hypothyroidism, in whom they observed a higher prevalence of depressive symptom (>60%) than in control (<30%) subjects. The present study documented a close correlation between QoL evaluated by means of the self-reported ThyPRO inventory and sleep disturbances evaluated subjectively by means of PSQI and ISI. In our experience, “anxiety” and “emotional susceptibility” were the items most significantly related with PSQI and ISI in both the DTC and control groups. In addition, the item “sexual life” was the one on which answers were most frequently missing. In the literature, this aspect has already been described [26, 48].

Several limitations need to be considered when interpreting the clinical significance of our results: (a) the current study was a cross-sectional investigation, which cannot fully establish a causal relationship between the studied factors; (b) our analyses relied mostly on self-report, which is subject to response bias; (c) no pre-surgery scores were available; (d) there are no data on the inventories used in the general Italian population; (e) the data cannot be extrapolated to DTC patients with still active thyroid malignancy; (f) a structured psychiatric interview was not performed; (g) no comparison between the sexes was made, owing to the low number of males enrolled.

As to the generalisability of our finding, we observed an acceptable response rate. We could not assess potential differences between responders, and non-responders, therefore the research participants may not reflect the entire study population. Additionally, it cannot be ruled out that some unmeasured characteristics (i.e., socioeconomic status and environmental factors) may have affected the response. Lastly, although the data analysis methods applied in this research met the study aims, further studies are necessary in order to better explore the cause-and-effect relationship between variables, and to examine confounding factors.

On the other hand, the inclusion of a comparison group gives strength to our study in at least two ways: enlarging the sample size, this increasing the power of the study; unravelling the physiological nature of the sleep disorders experienced by patients.

In conclusion, in DTC patients who are disease-free several years after diagnosis and in subjects who undergo thyroid surgery for benign pathology, abnormal sleep components and insomnia are similar. The ThyPRO questionnaire might be a useful instrument for detecting clinically relevant differences in QoL in DTC, as in benign thyroid pathology. The ThyPRO questionnaire correlates closely with sleep disturbances in all subjects. Recognising and treating sleep disturbances could improve QoL.

## Supplementary information

Supplementary information

Supplementary table

## Data Availability

The data that support the findings of this study are available upon reasonable request from the authors.
