# Supplementary material for: A comparative cross-sectional study on sleep quality in patients with a history of differentiated thyroid carcinoma and its correlation with quality of life
Source: Endocrine. 2021 Feb 3;73(2):347–57. doi: 10.1007/s12020-020-02591-z (PMC8263451; doi:10.1007/s12020-020-02591-z)
Supplement: Supplementary file 1 — Supplementary information [file 12020_2020_2591_MOESM1_ESM.pdf]

***SEZIONE 1°* (COMPILARE SEMPRE)**

Il/La sottoscritto/a________________________ nato/a ___________________ il __________________

in quanto paziente ☐ in quanto esercente legalmente la potestà ☐

per il paziente Sig./Sig.ra__________________________ nato/a ___________________ il ____________

ricevuta l'informativa di cui all'art. 13 del D. Lgs. 196/2003, nonché degli articoli 79 (Informativa da parte di organismi sanitari) e 81 (Prestazione del consenso) e preso atto dei diritti riconosciutigli dall'art. 7, del predetto decreto esprime il consenso al trattamento dei propri dati personali e sensibili nelle modalità e per le finalità sotto descritte e Regolamento Generale sulla Protezione dei Dati (RGDP) Regolamento UE 2016/679: (barrare con una crocetta la risposta)

AMBITO PER IL QUALE SI ESPRIME IL CONSENSO	FINALITÀ DEL TRATTAMENTO DEI DATI PERSONALI E SENSIBILI	SI	NO
INCONTRO ATTUALE CON LA STRUTTURA	ASSISTENZA SANITARIA		
	DIDATTICA (studenti universitari, specializzandi, dottorandi, assegnisti di ricerca,ecc.)		
	RICERCA SCIENTIFICA		
	ANALISI PER IL MIGLIORAMENTO		
DOSSIER SANITARIO ELETTRONICO (eventi clinici anteriori all'attuale)	CREAZIONE DEL DOSSIER SANITARIO ELETTRONICO		
	ASSISTENZA SANITARIA		
	DIDATTICA (studenti universitari, specializzandi, dottorandi, assegnisti di ricerca,ecc.)		
	RICERCA SCIENTIFICA		
	ANALISI PER IL MIGLIORAMENTO		
CONSERVAZIONE DEL MATERIALE BIOLOGICO (solo nel caso nell'episodio ne sia prevista la raccolta; vedi MODAOUHQA_0004)	ASSISTENZA SANITARIA		
	DIDATTICA (studenti universitari, specializzandi, dottorandi, assegnisti di ricerca,ecc.)		
	RICERCA SCIENTIFICA		
Attivazione del servizio Referti on-line (solo prestazioni ambulatoriali)			
Sportello Polifunzionale ASL 3 "Genovese", Servizi Territoriali , Autorità e Organi competenti			

Data_______________________________ Firma____

***SEZIONE 2°* - IN CASO DI INCAPACITA' DEL PAZIENTE**

Il sottoscritto Dott.____
dichiara che all'atto del ricovero, (il giorno ________________________alle ore _________________)
il/la Sig./Sig.ra ____
non può prestare il proprio consenso per impossibilità fisica, per incapacità di agire o per incapacità di intendere o di volere. I Testimoni sono:
Sig./Sig.ra ____
Sig./Sig.ra____

Data_______________ Firma del medico____

Stante l'incapacità del paziente di esprimere un consenso valido, il presente modulo viene firmato dal Sig/Sig.ra ____
identificato mediante documento valido Tipo___________________ N°____________________________
nella sua qualità di:
esercente legalmente la potestà ☐ prossimo congiunto ☐ familiare ☐ convivente ☐
responsabile della struttura presso cui dimora l'interessato ☐

che consente :

a chiunque ☐ a nessuno ☐ alle persone indicate nel box sul retro☐*

che la portineria renda noto ad eventuali richiedenti il luogo di ricovero **SI ❑ NO ❑**
Data_______________________________ Firma____

SEZIONE 3° **- IN CASO DI PAZIENTE MINORE**

Il sottoscritto Sig ____________________________ o la sottoscritta Sig.ra____________________________

esercenti la potestà genitoriale sul minore ____

nato a__________________________ il ________________ C.F.___________________________________

esprimono il proprio libero consenso al trattamento dei dati personali e sensibili del minore nelle modalità e per le finalità sotto descritte: (barrare con una crocetta la risposta)

AMBITO PER IL QUALE SI ESPRIME IL CONSENSO	FINALITÀ DEL TRATTAMENTO DEI DATI PERSONALI E SENSIBILI	SI	NO
INCONTRO ATTUALE CON LA STRUTTURA	ASSISTENZA SANITARIA		
	DIDATTICA (studenti universitari, specializzandi, dottorandi, assegnisti di ricerca,ecc.)		
	RICERCA SCIENTIFICA		
	ANALISI PER IL MIGLIORAMENTO		
DOSSIER SANITARIO ELETTRONICO (eventi clinici anteriori all'attuale)	CREAZIONE DEL DOSSIER SANITARIO ELETTRONICO		
	ASSISTENZA SANITARIA		
	DIDATTICA (studenti universitari, specializzandi, dottorandi, assegnisti di ricerca,ecc.)		
	RICERCA SCIENTIFICA		
	ANALISI PER IL MIGLIORAMENTO		
CONSERVAZIONE DEL MATERIALE BIOLOGICO (solo nel caso nell'episodio ne sia prevista la raccolta; vedi MODAOUHQA_0004)	ASSISTENZA SANITARIA		
	DIDATTICA (studenti universitari, specializzandi, dottorandi, assegnisti di ricerca,ecc.)		
	RICERCA SCIENTIFICA		
Attivazione del servizio Referti on-line (solo prestazioni ambulatoriali)			
Sportello Polifunzionale ASL 3 "Genovese", Servizi Territoriali , Autorità e Organi competenti			

Data_____________ Firma genitore_________________________________

Firma del minore (per espressione del proprio consenso) ______________________________

Consentono inoltre che la portineria renda noto ad eventuali richiedenti il luogo di ricovero SI ❑ NO ❑

SEZIONE 4° **- (COMPILARE SEMPRE) *Consenso alla comunicazione riguardo lo stato di salute**

a nessuno ❑ alle seguenti persone sotto indicate ❑ al fiduciario nominato ❑**

Cognome Nome	Grado di parentela o altro	Recapito telefonico
al medico curante: Dott……………………………………………………		

che la portineria renda noto ad eventuali richiedenti il luogo di ricovero **SI** ❑ **NO** ❑

Data____________________ Firma____

SEZIONE 5° **-** ** **Nomina del fiduciario**, ai sensi del Regolamento Regionale 9 .4.2014 N. 2 "Designazione del Fiduciario", si allega Nomina Regionale o allegato MODAZHQA_0016
